# High prevalence of sacroiliitis and early structural changes in the sacroiliac joint in children with enthesitis-related arthritis: findings from a tertiary centre in Hong Kong

**DOI:** 10.1186/s12969-023-00825-8

**Published:** 2023-05-03

**Authors:** Oi Man Chan, Billy Ming-Hei Lai, Agnes Sze-Yin Leung, Ting Fan Leung, Assunta Chi-Hang Ho

**Affiliations:** 1 Department of Paediatrics, Faculty of Medicine, The Chinese University of Hong Kong, Hong Kong, Hong Kong SAR; 2 Department of Diagnostic and Interventional Radiology, Prince of Wales Hospital, Hong Kong, Hong Kong SAR; 3 Department of Paediatrics, Prince of Wales Hospital, Hong Kong, Hong Kong SAR

**Keywords:** Juvenile idiopathic arthritis, Enthesitis-related arthritis, Sacroiliitis, Sacroiliac joint

## Abstract

**Background:**

Epidemiological studies have demonstrated a wide, unexplained disparity in the prevalence of juvenile idiopathic arthritis (JIA) subtypes depending on geographical location, ethnicity and other factors. Enthesitis-related arthritis (ERA) is more prevalent in Southeast Asia. Axial involvement in ERA patients is increasingly recognised to occur early in the disease course. Inflammation in the sacroiliac joint (SIJ) observed on MRI seems highly predictive of subsequent structural radiographic progression. The resulting structural damage can have significant impacts on both functional status and spinal mobility. This study aimed to evaluate the clinical characteristics of ERA in a tertiary centre in Hong Kong. The primary objective of the study was to provide a comprehensive description of the clinical course and radiological findings of the SIJ among ERA patients.

**Method:**

Paediatric patients diagnosed with JIA attending the paediatric rheumatology clinic from January 1990 to December 2020 were recruited from our registry based at the Prince of Wales Hospital.

**Results:**

In our cohort, 101 children were included. The median age of diagnosis was 11 years, interquartile range (IQR) 8-15 years. The median follow-up duration was 7 years (IQR 2–11.5 years). ERA was the most prevalent subtype (40%), followed by oligoarticular JIA (17%).

Axial involvement was frequently reported in our cohort of ERA patients. 78% demonstrated radiological evidence of sacroiliitis. Among those, 81% had bilateral involvement. The median duration from disease onset to confirmation of radiological sacroiliitis was 17 months (IQR 4-62 months). Among the ERA patients, 73% had structural changes of the SIJ. Alarmingly, 70% of these patients had already developed radiological structural changes when sacroiliitis was first detected on imaging (IQR 0-12 months). Erosion was the most common finding (73%), followed by sclerosis (63%), joint space narrowing (23%), ankylosis (7%) and fatty change (3%). The duration from symptom onset to diagnosis was significantly longer in ERA patients with SIJ structural changes (9 vs 2 months, *p* = 0.009), comparing with those without.

**Conclusion:**

We found that a high proportion of ERA patients had sacroiliitis and a significant number of them had radiological structural changes during early disease. Our findings illustrate the importance of prompt diagnosis and early treatment in these children.

## Background

Juvenile idiopathic arthritis (JIA) is the most common inflammatory arthritis in children. The International League of Associations for Rheumatology (ILAR) defined it as chronic arthritis lasting for six weeks or more of unknown origin starting before the age of 16 years. The ILAR classification consists of seven mutually exclusive categories defined on the basis of clinical and laboratory features at presentation in the first 6 months of illness [1].

Consolaro et al. showed in an observational study, which included 9081 patients from 49 countries, that enthesitis-related arthritis (ERA) (30%, 113 of 379) and systemic arthritis (33%, 125 of 379) were more common in southeast Asia, whereas oligoarthritis (57%, 1360 of 2400) was more prevalent in southern Europe and rheumatoid factor (RF)-negative polyarthritis (32%, 165 of 523) was more frequent in North America than in other areas, demonstrating a remarkable variability in the prevalence of JIA subtypes [2]. We are aware that ERA is more commonly observed in the Chinese population, likely due to the high prevalence of human leukocyte antigen B27 (HLA-B27) in this demographic [3, 4].

ERA patients are generally considered having less favourable outcomes than patients with other JIA subtypes [5]. In a retrospective study in Taiwan, sacroiliitis was a predictor for persistent active disease in ERA patients [6]. Additionally, a Singaporean study done by Arkachaisri [7] showed that among methotrexate-treated sacroiliitis patients, 85.3% failed to respond requiring anti-tumour necrosis factor (aTNF), as compared to 63.2% patients without axial disease. ERA is part of the spondyloarthropathy (SpA) spectrum [8]. SpA can be subdivided into peripheral SpA and axial SpA, the latter of which can further be subdivided into radiographic and non-radiographic categories. In ERA, it is increasingly recognised that axial involvement can occur early in the disease course [9]. Dougados showed that inflammation in the sacroiliac joint observed on MRI is highly predictive of subsequent structural radiographic progression in adult [10]. Moreover, structural damage in the SIJ might have significant impacts on both functional status and spinal mobility of axial SpA patients [11]. This study aimed to examine the clinical characteristics of ERA in a tertiary centre in Hong Kong. The primary objective of the study was to provide a comprehensive description of the clinical and radiological findings of ERA patients. Furthermore, we compared the similarities and differences between the ERA patients in this cohort and those from Southeast Asia and Western countries.

## Methods

Paediatric patients <18years of age who fulfilled the ILAR criteria for diagnosis and classification of JIA attended the paediatric rheumatology clinic from January 1990 to December 2020 were recruited from our registry based at the Prince of Wales Hospital [12]. Prince of Wales Hospital is a teaching hospital of the Chinese University of Hong Kong and a tertiary referral centre located in the New Territories East region. The diagnosis of ERA was made by the presence of arthritis and enthesitis or the presence of either arthritis or enthesitis with two or more of the following criteria: SIJ tenderness or inflammatory lumbosacral pain, presence of HLA-B27, onset of arthritis in a boy after the age of six, occurrence of acute anterior uveitis and family history of HLA-B27 associated disease such as ankylosing spondylitis [1]. For those who had presented before the ILAR classification was established, the American College of Rheumatology (ACR) classification of juvenile rheumatoid arthritis and the European Alliance of Associations for Rheumatology (EULAR) classification of juvenile chronic arthritis were used instead. Other inclusion criteria included age below 18 years when they were first assessed at our centre, having more than one clinic visits after the intake assessment, and having data trackable from the electronic health record management system. Exclusion criterion was inadequate information to validate the accuracy of data.

Data including demographics, clinical assessments, laboratory findings, imaging results and medication history were collected from medical records retrospectively for patients who were given the diagnosis prior to 2005 and then prospectively after enrolment. Disease onset was defined by the onset of symptoms. For ERA, symptoms relevant were those that were suggestive of enthesitis, peripheral arthritis and inflammatory back pain. Disease activity in the sacroiliac joints (SIJs) was assessed in the history taking enquiring about lower back, hip or buttock pain, by direct palpation of the SIJs on examination and via specific manoeuvrers such as Patrick’s test. We also included the Bath Ankylosing Spondylitis Disease Activity Index (BASDAI) and Bath Ankylosing Spondylitis Functional Index (BASFI) to assess disease activity and functional impairment in ERA patients [13]. The follow-up duration was calculated based on the length of time between the initial intake assessment and the last recorded clinc visit.

**Fig. 1:**
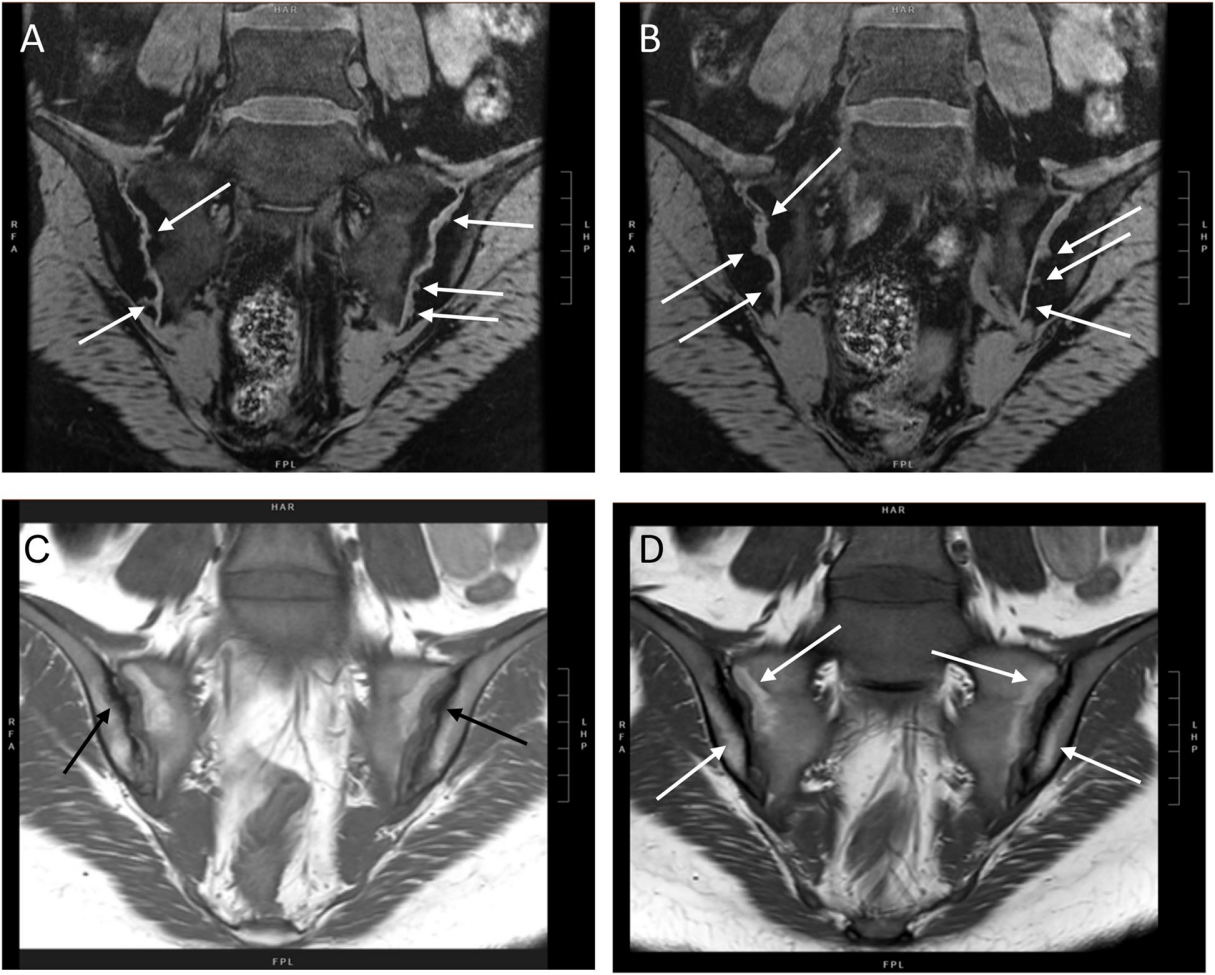
Structural damage lesions. **A** and **B** Coronal SPGR FS (Spoiled Gradient-Recalled Echo with Fat Suppression) showing multiple periarticular erosions (white arrows) along both sacroiliac joints. **C** and **D** Coronal T1 sequence showing sclerosis (hypointense signal, black arrows) and fatty changes (hyperintense signal, white arrows) over bilateral sacroiliac joints

**Fig. 2:**
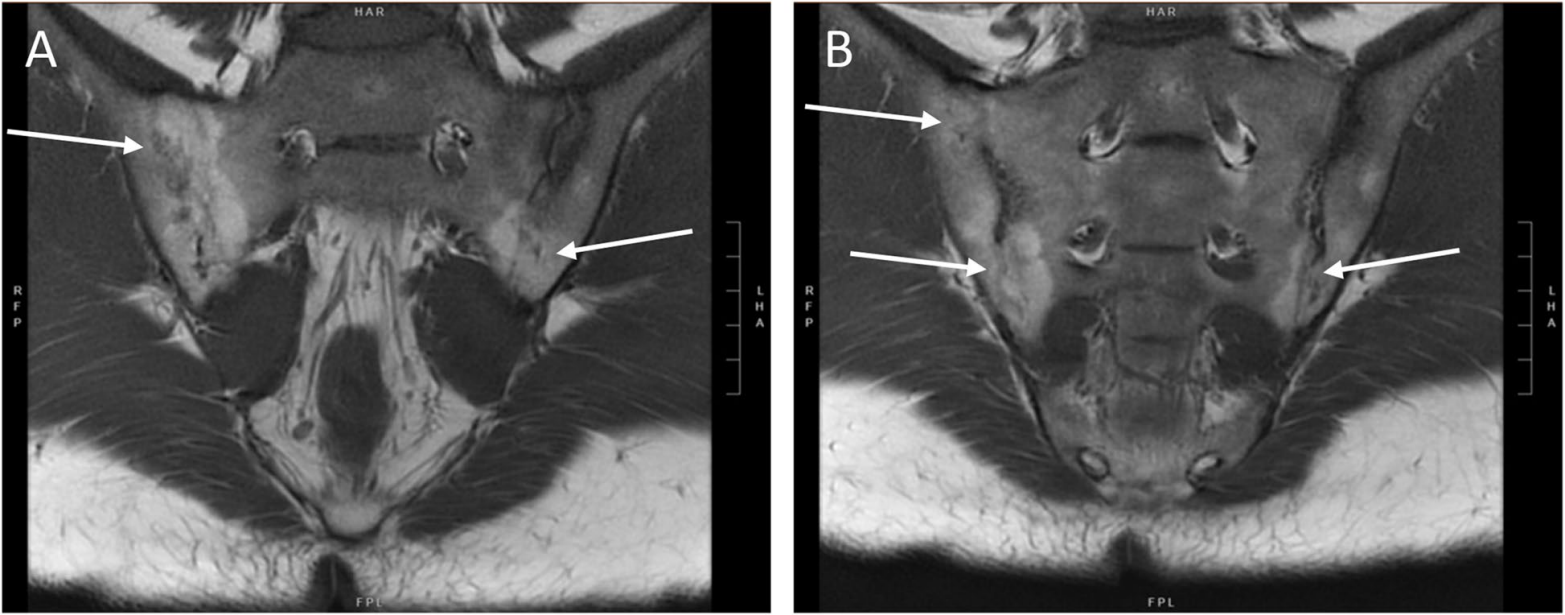
Ankylosis. **A** and **B** Coronal T1 sequence showing partial osseous fusion (white arrows), more severe at the right sacroiliac joints

**Fig. 3:**
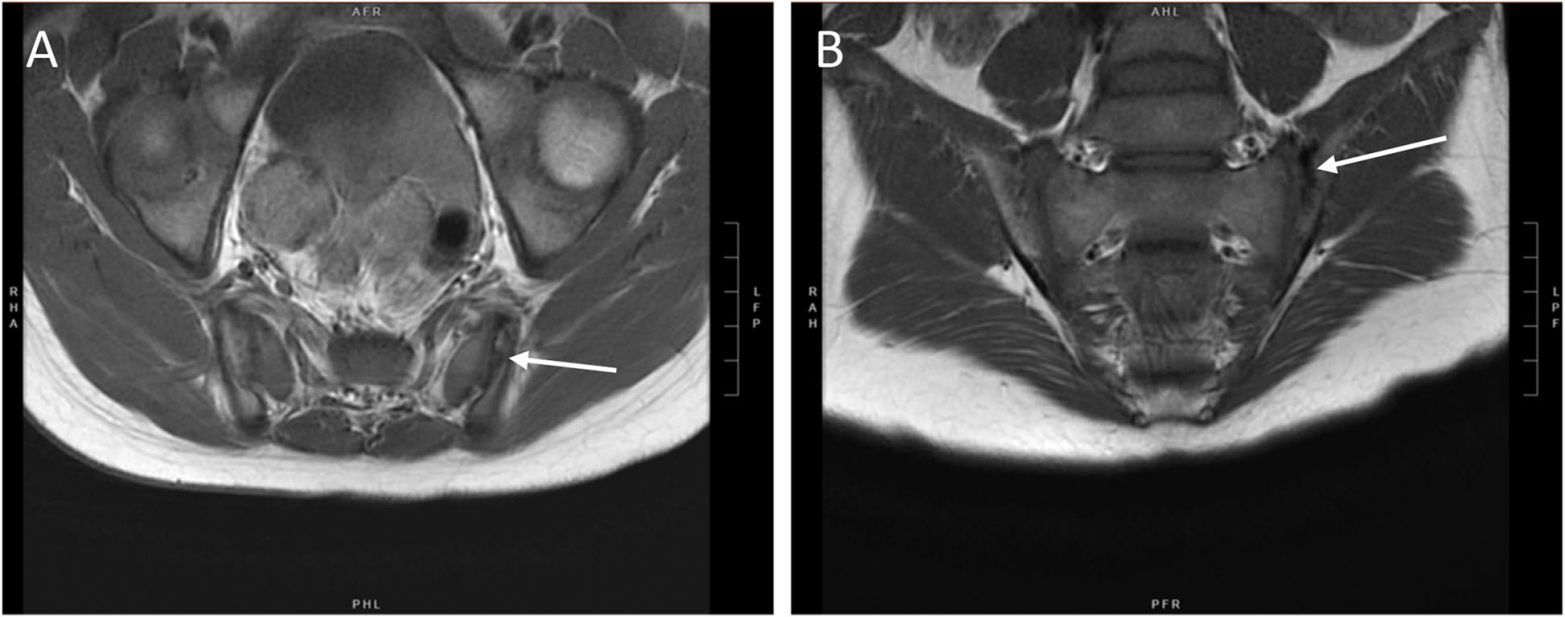
Early structural damage lesions in a 10-year-old boy. **A** Axial T1 sequence showing joint surface irregularity (white arrow) at the left sacroiliac joint, representing erosion. **B** Coronal T1 sequence showing sclerotic areas (hypointense signal, white arrow) in the left iliac bone

Imaging of the SIJ was not routinely done unless there was any symptom or sign suggestive of sacroiliitis. Radiographs would typically be obtained first. Alternatively, the treating physician might choose to arrange for magnetic resonance imaging (MRI) if there was a strong clinical suspicion of disease activity and chronicity in the SIJs. Sacroiliitis on radiographs was defined according to the modified New York criteria [14]. Active sacroiliitis on MRI was identified by the presence of bone marrow oedema, whereas structural changes in the SIJ such as erosion, sclerosis, fat deposition and ankylosis were assessed based on the Assessment of SpondyloArthritis international Society (ASAS) criteria as shown in Figs. 1, 2 and 3 [15]. The imaging was reviewed by consultant radiologists with more than 8 years of experience in paediatric imaging. The study was approved by the Joint Chinese University of Hong Kong – New Territories East Cluster Clinical Research Ethics Committee (CREC Ref No.: 2020.305).

### Statistical analysis

Results were expressed as percentages for categorical variables, mean with standard deviation or median with interquartile range for continuous variables according to the normality of the data. Chi-squared test, Fisher’s exact test, two sided independent-samples t-test and Mann–Whitney U test were applied to compare differences between groups where appropriate. Statistical analyses were performed using SPSS version 25.0 (IBM Corp., Armonk, NY, USA). A *p* value < 0.05 was considered to be statistically significant.

## Results

A total of 105 patients were identified from the local registry, of which 101 were eligible for analysis. One patient was excluded due to the unavailability of data on the electronic health record management system (*n* = 1) and those with alternative diagnoses other than JIA (*n* = 3) were also excluded.

In our cohort (Table 1), ERA was the most prevalent subtype (*n* = 41, 40%), followed by oligoarticular JIA (*n* = 17, 17%). There were ten patients in the undifferentiated group. Among the three patients who presented with ERA symptoms, it was observed that either they had a family history of psoriasis or later developed psoriasis clinically.

**Table 1:** JIA subtypes and demographics

	Total (*n* = 101)	ERA (*n* = 41)
JIA subtypes, n (%)		
Enthesitis-related arthritis	41 (40)	
Oligoarticular JIA	17 (17)	
Polyarticular JIA (rheumatoid factor negative)	12 (12)	
Polyarticular JIA (rheumatoid factor positive)	7 (7)	
Systemic arthritis	10 (10)	
Psoriatic arthritis	4 (4)	
Undifferentiated arthritis	10 (10)	
Age of diagnosis in years, median (IQR)	11 (8–15)	12 (10.3–15)
Male, n (%)	60 (59)	35 (85)
Chinese, n (%)	97 (96)	41 (100)
Follow-up duration in years, median (IQR)	7 (2–11.5)	7 (2.5–13)

*JIA* Juvenile idiopathic arthritis (JIA), *ERA* Enthesitis-related arthritis, *N* Number, *IQR* Interquartile range

Demographics of the cohort was also shown in Table 1. There was a male preponderance with a male to female ratio of 1.46:1. The majority of the cohort were Chinese (96%). The median age of diagnosis was 11 years, with an interquartile range (IQR) of 8-15 years. With a median follow-up duration of 7 years (IQR 2–11.5 years), 30 patients were lost to follow-up leaving 71 patients (70%) remained under our care.

In patients with ERA, axial involvement was frequently reported (Table 2). Human leukocyte antigen B27 (HLA-B27) was positive in 95%. The median age of disease onset was 11 years (10-14 years). Imaging of the SIJ was performed in 98% of ERA patients, 88% had radiographs taken, 71% had MRI done and 61% had both. A significant proportion of children (*n* = 32, 78%) demonstrated radiological evidence of sacroiliitis. Among those, 81% had bilateral involvement. The median duration from disease onset to confirmation of radiological sacroiliitis was 17 months (4-62 months).

**Table 2:** Clinical and radiological characteristics of ERA patients

	Total (*n* = 41)
HLA-B27, n (%)	39 (95)
Family history in 1^st^ degree relative of HLA-B27 associated disease, n (%)	6 (15)
Disease onset in years, median (IQR)	11 (10–14)
Age of diagnosis in years, median (IQR)	12 (10.3–15)
Imaging modalities of SIJ, n (%)	40 (98)
Radiograph, n (%)	36 (88)
MRI, n (%)	29 (71)
CT, n (%)	1 (2)
Sacroiliitis, n (%)	32 (78)
Duration from disease onset to sacroiliitis in months, median (IQR)	17 (4–62)
Sacroiliac joint structural changes, n (%)	30 (73)
Duration from disease onset to SIJ structural changes in months, median (IQR)	25 (14–64)
Duration from diagnosis of sacroiliitis to SIJ structural changes in months, median (IQR)	0 (0–12)
Erosions, n (%)	22 (54)
Sclerosis, n (%)	19 (46)
Joint space narrowing, n (%)	7 (17)
Ankylosis, n (%)	2 (5)
Fatty change, n (%)	1 (2)

*ERA* Enthesitis-related arthritis, *N* Number, *HLA-B27* Human leukocyte antigen B27, *IQR* Interquartile range, *SIJ* Sacroiliac joint, *MRI* Magnetic resonance imaging, *CT* Computer tomography

An overwhelming proportion of ERA patients with sacroiliitis had structural changes of the SIJ (*n* = 30, 73%). Erosions was the most common finding (73%), followed by sclerosis (63%), joint space narrowing (23%), ankylosis (7%) and fatty change (3%). Alarmingly, among those patients with SIJ structural defect, 70% (*n* = 21) were already noted to have those changes at the time of first imaging showing sacroiliitis (IQR 0-12 months). When comparing the two groups of ERA patients with and without structural changes of the SIJ (Table 3), we identified that the duration from disease onset to diagnosis was significantly longer in those with structural changes (9 vs 2 months, *p* = 0.009).

**Table 3:** ERA patients’ clinical characteristics by the presence of SIJ structural change

	**SIJ structural change (*n* = 30)**	**No structural change (*n* = 11)**	***P* value**
Male, n (%)	26 (86.7%)	9 (81.8%)	0.651
Age of onset in years, median (IQR)	12 (10–15)	10 (9–11)	0.064
Buttock pain at presentation, n (%)	3 (11.1%)	2 (20%)	0.597
Low back pain at presentation, n (%)	9 (33.3%)	0 (0%)	0.079
Hip involvement within 1^st^ 6 months, n (%)	10 (35.7%)	4 (40%)	1
Ankle involvement within 1^st^ 6 months, n (%)	1 (3.6%)	2 (20%)	0.164
Duration from onset to diagnosis in months, median (IQR)	9 (4–17.5)	2 (1–5)	*0.009
Duration from diagnosis to 1^st^ MRI of SIJ in months, median (IQR)	2.5 (0–42.8)	15 (2–128.5)	0.447
Duration of disease, median (IQR)	12.4 (7.4–19.3)	6.9 (4.3–19.6)	0.204
Abnormal mSchober at diagnosis, n (%)	7 (35%)	0 (0%)	0.137
CRP at presentation (mg/L), median (IQR)	14.6 (1.75–35.5)	7.9 (2.9–22.6)	0.645
ESR at presentation (mm/hr), mean (SD)	39.5 (29.6)	46.1 (17.5)	0.534
Latest BASDAI, median (IQR)	1.2 (0.2–2.2)	1.16 (0.31–2.25)	0.864
Latest BASFI, median (IQR)	0.23 (0.05–0.89)	0.35 (0.07–0.85)	0.845
mSchober at last visit (cm), median (IQR)	6 (5–6)	7 (5–7)	0.227
Biologic use, n (%)	11 (36.7%)	2 (18.2%)	0.451

*SIJ* Sacroiliac joint, *N* Number, *IQR* Interquartile range, *SD* Standard deviation, *MRI* Magnetic resonance imaging, *mSchober* Modified Schober test, *CRP* C-reactive protein, normal range (< 9.9 mg/L), *ESR* Erythrocyte sedimentation rate, normal range (2-19 mm/hr), *BASDAI* Bath ankylosing spondylitis disease activity index, *BASFI* Bath ankylosing spondylitis functional index

Regarding to past treatment for the whole cohort (Table 4), conventional disease-modifying anti-rheumatic drugs (cDMARDs) were prescribed in 59% patients and 20% had tried two or more. Tumour necrosis factor inhibitors (aTNF) were given to 21% patients. Anti- interleukin-6 (anti-IL6) was used in two patients, anti-interleukin-1 (anti-IL1) in one patient and janus kinase inhibitors (JAKi) in two patients. A considerable proportion (*n* = 42, 41%) had never received any immunomodulators. Among the patients who were still under active care, 32% patients were taking cDMARDs and 20% were on anti-TNFs.

**Table 4:** Current and past medications

	Total (*n* = 101)	ERA (*n* = 41)	*P* value
Past treatment, n (%)	60 (59)	29 (71)	
cDMARDs	60 (59)	29 (71)	0.206
Anti-TNF	21 (21)	12 (29)	0.278
Anti-IL6	2 (2)	0 (0)	1.0
Anti-IL1	1 (1)	0 (0)	1.0
JAKi	2 (2)	0 (0)	1.0
Current follow-up, n (%)	71 (70)	31 (76)	
Active treatment, n (%)	34 (48)	14 (45)	0.8
cDMARDs	23 (32)	7 (23)	0.132
anti-TNF	14 (20)	8 (26)	0.762

*N* Number, *ERA* Enthesitis-related arthritis, *cDMARD* Conventional disease-modifying anti-rheumatic drug, *Anti-TNF* Tumour necrosis factor inhibitor, *Anti-IL6* Interleukin-6 inhibitor, *Anti-IL1* Interleukin-1 inhibitor, *JAKi* Janus kinase inhibitor

Among the ERA patients (*n* = 41), 71% patients were treated with cDMARDs, with 15% being tried on two or more. Anti-TNFs were used in 29% ERA patients with 5% being treated with two. Among those who were still under active care (*n* = 31, 76%), seven patients were on cDMARDs and eight patients were on anti TNFs. A patient was being managed with the combination of both cDMARD and anti-TNF. More than half of the patients (55%) were not on any treatment besides non-steroidal anti-inflammatory drugs (NSAIDs).

## Discussion

The most common JIA subtype was ERA in our cohort, with male preponderance and high HLA-B27 positivity. This is similar to what had been reported by Shih et al. [6] and a more recent survey done by Consolaro et al. [2]. Gmuca [16] suggested that HLA-B27 positivity was associated with sacroiliitis and higher disease activity at disease onset. This might explain why axial involvement was strikingly prevalent in our ERA patients. Our findings of 78% having radiological evidence of sacroiliitis is much higher than that reported in the Taiwanese study (16%), in which sacroiliitis was defined as having either clinical symptom or radiological changes. Other symptoms or signs suggestive of sacroiliitis, namely buttock pain, SIJ tenderness on direct palpation or elicited by stress test were not mentioned. The proportion of patients with either clinical or radiological features was not reported. Our result is more consistent with the French cohort, in which, 63% had axial symptoms and 47% developed sacroiliitis at a median time of 2.6 and 5.3 years respectively [9]. On the other hand, Li [17] found 88.5% ERA patients had sacroiliitis at diagnosis in a retrospective study done in Shanghai. Together, these results suggested that axial involvement might occur early in the disease course and more frequently than expected in children with ERA.

The use of MRI to image the sacroiliac joints has increased tremendously in the last decade, particularly in evaluating patients with ERA, as clinical symptoms and examination alone had low predictive value for sacroiliitis [5, 18, 19]. Recently, Weiss reported a wide range of discrepancies in interpreting active and chronic sacroiliitis on MRI by comparing local radiology reports and those reviewed by experienced musculoskeletal paediatric radiologists as reference standard. It illustrated a substantial variation exists in interpretation of inflammatory and structural changes in the SIJs of children [20]. Besides, there is no clear guidance on the timing and frequency of MRI to aid the assessment and monitoring of sacroiliitis. This could impact on the pick-up rate of sacroiliitis and potential structural changes depending on the level of suspicion of the treating clinicians [21].

In addition, we reported a significant portion (*n* = 30, 73%) of our ERA patients had radiological structural changes of SIJs, among which (*n* = 21, 70%) were already present at the time of first imaging detected sacroiliitis. We identified that the duration from disease onset to diagnosis was significantly longer in children with SIJ structural changes (*p* = 0.009) than in those without. Our findings illustrate the importance of prompt diagnosis and early treatment in children with ERA. Early control of the inflammation could possibly prevent worsening structural changes to the sacroiliac joint in the long run. This is supported by a recent study in adults with axial spondyloarthritis done by Walter [22], which showed a larger number of patients achieved regression of erosion in the sacroiliac join on MRI with versus without etanercept (23.1% vs 2.9%; *p* = 0.01).

Our cohort demonstrated an overwhelming proportion of ERA patients having history of sacroiliitis (*n* = 32, 78%) and radiological structural changes of SIJs (*n* = 30, 73%) and yet only 12 patients (29%) had ever been on biologics. It is concerning that the use of biologics in treating children with JIA in our locality might be affected by the reimbursement policy, which is based on household income. According to the 2019 ACR/Arthritis Foundation treatment guideline for JIA, biologics are recommended after trial of NSAIDs in active sacroiliitis [23]. The local funding practice does not take sacroiliitis, despite its prevalence, as a stand alone entity into consideration in treatment of ERA. It remains speculative if parental or physician’s decision on starting biologics in our locality might have been affected by financial constraints.

This study has several limitations. Firstly, patients were recruited from a single tertiary centre. It is important to note that the majority, if not all, of the JIA patients in Hong Kong are under the care of tertiary centres. Also, the Prince of Wales Hospital is among the few hospitals in Hong Kong that provides paediatric rheumatology services. Therefore, our findings should be representative of the New Territories East region. However, to confirm the generalisability of our results to the entire city, future studies conducted across the territory would be necessary. The time frame of the study spanned 30 years. Some important information such as enthesitis assessment, disease activity and functional impairment measures were not routinely documented in the early years. Additionally, the timing and frequency of imaging were not standardised, as well as the fact that reporting of radiological findings is based on individual expertise and experience. MRIs of SIJs were arranged depending on clinical suspicion. There was always time-lag between physician’s request and actual imaging. Extra-articular manifestations including enthesitis, uveitis and gut inflammation have not been studied thoroughly. Lamot demonstrated that faecal calprotectin concentration was notably higher in ERA patients with active disease and showing MRI signs indicative of SIJ inflammation, which contributes to the increasing evidence that supports the clinical association between gut inflammation and axial spondyloarthritis, both in adults and children [24]. Subclinical gut inflammation has not been addressed in this study.

## Conclusion

We described the clinical characteristics of ERA patients at a tertiary centre in Hong Kong. We found that a high proportion of children with ERA had sacroiliitis and a significant number of them had radiological structural changes during early disease. Our findings illustrate the need of better evaluation of the SIJs in children with ERA, as well as the importance of prompt diagnosis and early treatment.

## Data Availability

The data that support the findings of this study are available on request from the corresponding author, Assunta CH Ho. Patient-related data not included in the paper might be subject to patient confidentiality.

## Abbreviations

ERA: Enthesitis-related arthritis
JIA: Juvenile idiopathic arthritis
SIJ: Sacroiliac joint
ILAR: International League of Associations for Rheumatology
RF: Rheumatoid factor
HLA-B27: Human leukocyte antigen B27
aTNF: Anti-tumour necrosis factor
SpA: Spondyloarthritis
ACR: American College of Rheumatology
EULAR: European League Against Rheumatism
BASDAI: Bath Ankylosing Spondylitis Activity Index
BASFI: Bath Ankylosing Spondylitis Function Index
MRI: Magnetic resonance imaging
CT: Computer tomography
ASAS: Assessment of SpondyloArthritis international Society
cDMARD: Conventional disease-modifying anti-rheumatic drugs
Anti-IL6: Anti-interleukin-6
Anti-IL1: Anti-interleukin-1
JAKi: Janus kinase inhibitor
NSAIDs: Non-steroidal anti-inflammatory drugs
N: Number
IQR: Interquartile range
SD: Standard deviation
